# A Missense Mutation in *OPA1* Causes Dominant Optic Atrophy in a Chinese Family

**DOI:** 10.1155/2019/1424928

**Published:** 2019-11-03

**Authors:** Shaoyi Mei, Xiaosheng Huang, Lin Cheng, Shiming Peng, Tianhui Zhu, Liang Chen, Yan Wang, Jun Zhao

**Affiliations:** ^1^Shenzhen Eye Hospital Affiliated to Jinan University, Shenzhen Eye Institute, Shenzhen, Guangdong 518040, China; ^2^School of Optometry Affiliated to Shenzhen University, Shenzhen, Guangdong 518040, China; ^3^State Key Laboratory of Ophthalmology, Zhongshan Ophthalmic Center, Sun Yat-sen University, Guangzhou 510060, China

## Abstract

**Background:**

To investigate the genetic causes and clinical characteristics of dominant optic atrophy (DOA) in a Chinese family.

**Methods:**

A 5-generation pedigree of 35 family members including 12 individuals affected with DOA was recruited from Shenzhen Eye Hospital, China. Four affected family members and one unaffected family member were selected for whole exome sequencing. Sanger sequencing was used to confirm and screen the identified mutation in 18 members of the family. The disease-causing mutation was identified by bioinformatics analysis and confirmed by segregation analysis. The clinical characteristics of the family members were analyzed.

**Results:**

A heterozygous missense mutation (c.1313A>G, p.D438G) in *optic atrophy 1* (*OPA1*) was identified in 10 individuals affected with DOA in this family. None of the unaffected family members had the mutation. Patients in this family had vision loss since they were children or adolescence. The visual acuity decreased progressively to hand movement, except for one patient (IV-12) who had relatively good vision of 20/30 and 20/28. The fundus typically manifested as optic disc pallor. The visual fields, optical coherence tomography, and visual evoked potential suggested variable degree of abnormality in patients. Patients who had a history of cigarette smoking and alcohol drinking had more severe clinical manifestations.

**Conclusions:**

Our results suggest that the p.D438G mutation in *OPA1* causes optic atrophy in this family. The patients who carried the mutation demonstrated heterogeneous clinical manifestations in this family. This is the first report on the c.1313A>G (p.D438G) mutation of *OPA1* in a Chinese family affected with DOA.

## 1. Introduction

Optic atrophy is an irreversible vision loss disease due to primary death of retinal ganglion cell (RGC) and its axons. Dominant optic atrophy (DOA), also known as Kjer's optic neuropathy [1], is regarded as a representative of one of the two classic paradigms of mitochondrial dysfunction in inherited optic neuropathies, with Leber's hereditary optic neuropathy (LHON) being the other [2]. The incidence of DOA is estimated to be 1 : 50,000 with prevalence as high as 1 : 10,000 in the Danish population [3]. Although the disease has a high penetrance (88%), severity and progression of DOA are extremely variable even within the same family [4].

DOA has an insidious onset, and visual loss usually occurs in the first two decades of life [5]. The disease is characterized by a bilateral degeneration of optic nerves, primarily affecting the RGCs and their axons forming the optic nerve. The patients suffer moderate-to-severe visual loss, associated with central or paracentral visual field deficits and color vision defects. Ophthalmic examinations disclose on fundoscopy optic disc pallor or atrophy, related to the RGC death [6]. Some DOA patients harbor extraocular multisystemic features, named as dominant optic atrophy plus syndromes (DOA+), such as sensorineural deafness, or less commonly chronic progressive external ophthalmoplegia, ataxia and/or myopathy [7], or nonsyndromic, idiopathic Parkinson's disease [8].

DOA is mainly caused by mutations in *OPA1*, a nuclear gene located on chromosome 3q, which encodes a mitochondrial dynamin-related GTPase protein that appears to be involved in mitochondrial membrane biogenesis and stabilization of membrane integrity [6, 9–11]. The mutation of *OPA1* will lead to mitochondrial dysfunction and subsequently apoptosis of the retinal ganglion cells. Therefore, damage of the papillomacular bundle in DOA is the consequence of the mitochondrial dysfunction caused by *OPA1* mutation [12]. DOA and LHON are difficult to be differentiated especially when the patients are in childhood, while genetic testing can assist in diagnosis of the disease [13].

In this study, we described a Chinese family affected with DOA caused by a heterozygous missense mutation in *OPA1*. Ten affected patients had visual loss, pale optic disc, lower amplitude and longer latency of visual evoked potential (VEP) test, thinner retinal nerve fiber layer (RNFL), and narrow visual fields.

## 2. Methods

### 2.1. Patients and Ophthalmological Examinations

A 5-generation pedigree of 35 family members including 12 individuals affected with DOA was recruited from Shenzhen Eye Hospital, China. All of the family members are Han Chinese. DOA was diagnosed according to the following criteria [6]: (1) all patients have bilateral and symmetric moderate-to-severe progressive visual loss during their early childhood and typically occurring in the context of a family history of DOA; (2) fundoscopy reveals full pallor or temporal pallor of optic disc on both eyes; (3) central or paracentral scotoma and some patients may have extensive visual field defects (VFD); (4) optical coherence tomography (OCT) discloses nonspecific thinning of RNFL especially at the temporal part but a normal morphology of the photoreceptor layers; (5) abnormal VEP; (6) excluding optic atrophy induced by other congenital malformations from the eye or other systems and trauma; and (7) molecular genetic analysis allows patients to be adequately diagnosed.

The family members underwent the following ophthalmological examinations including best corrected visual acuity (BCVA), color testing, anterior segment exam, fundoscopy, visual field, OCT, VEP, and intraocular pressure (IOP). Optic disc, RNFL thickness, and macula were examined using OCT in 10 eyes of 5 affected patients (DOA group) and 6 eyes of 3 unaffected members (control group). The thickness of superior, inferior, temporal, and nasal quadrants of RNFL was analyzed, respectively. Visual field and VEP were measured with different strategies according to patients' vision. Those with vision above finger counting were measured by dynamic balance test strategy in visual field and pattern visual evoked potential (P-VEP). Patients who had vision of finger counting or hand movement were measured by central low vision test strategy in visual field and flash visual evoked potential (F-VEP).

The study protocol was approved by the Independent Ethics Committee of Shenzhen Eye Hospital in accordance with the tenets of the Declaration of Helsinki. Written informed consent was obtained from all study participants.

### 2.2. Genomic DNA Extraction and Whole Exome Sequencing

Genomic DNA was extracted from blood samples of 18 family members (10 affected individuals: III-1, III-3, III-9, III-10, IV-3, IV-5, IV-8, IV-10, IV-12, and V-2; 8 unaffected members: III-5, IV-1, IV-4, IV-6, IV-13, V-1, V-3, and V-6). The final concentration of DNA samples was diluted to 50 ng/*μ*L. Whole exome sequencing (WES) [14] was performed for 5 family members, including four affected individuals (i.e., V-2, III-3, III-9, and IV-5) and one unaffected member (i.e., IV-1) by the Novogene company (Beijing, China). For each sample, 0.4 *μ*g of genomic DNA was randomly sonicated into fragments and used to construct a paired-end sequencing library. Exome capture was performed using a SureSelect Human All Exon v6+UTR capture kit (Agilent Technologies, Santa Clara, CA). Each sample underwent 2 × 150 bp paired-end sequencing on a HiSeq 4000 Next-Generation Sequencing system (Illumina, San Diego, CA). The sequenced reads were aligned to the human reference genome (hg19) using the Burrows–Wheeler Aligner (BWA) [15]. Sequence variants were called using Samtools and GATK [16, 17] and annotated with ANNOVAR [18]. The transcript variant NM_015560 was used to denominate the mutation of *OPA1*.

### 2.3. Variant Filtering and Mutation Verification

Sequence variants were filtered based on the following criteria: (1) novel or rare in population database: minor allele frequency <1% in Exome Aggregation Consortium (ExAC); (2) most likely pathogenic: pathogenicity score of at least “possibly damaging” or “disease causing” after annotation by Sorts Intolerant From Tolerant (SIFT), Polymorphism Phenotyping v2 (Polyphen-2), and MutationTaster; (3) evidence of evolutionary conservation (GERP score > 0); (4) Combined Annotation Dependent Depletion (CADD score > 15); and (5) heterozygotes consistent with the dominant inheritance pattern.

The identified mutation in *OPA1* was verified using polymerase chain reaction (PCR) and Sanger sequencing. The primer sequences used in PCR and Sanger sequencing were: (1) forward primer: 5′-GCCATCATACTGTGTATTCAAGG-3′ and (2) reverse primer: 5′-GTGAGCCTGGTTTCCTTCAG-3′.

### 2.4. Statistical Analysis

Statistical analysis was performed using the SPSS 24.0 software (SPSS Inc, Chicago, IL). Since the RNFL thickness in the nasal quadrant followed normal distribution, the data were presented as mean ± standard deviation. RNFL thickness in this quadrant was compared between patients with DOA and unaffected controls using *t*-test. While the RNFL thickness in the superior, inferior, and temporal quadrants did not follow normal distribution, the data were presented as median (quartile 1 and quartile 3). RNFL thickness in three quadrants was compared between patients with DOA and unaffected controls using Mann–Whitney *U* test. The BCVA was compared between smokers and nonsmokers or between alcohol drinkers and nondrinkers in patients of this family using the Student's *t*-test. The logMAR figures “2.30” and “1.85” were applied for those subjects tested as “HM” and “FC,” respectively [19]. *P* < 0.05 was considered statistically significant.

## 3. Results

### 3.1. DOA Pedigree and Patients

**Figure 1:**
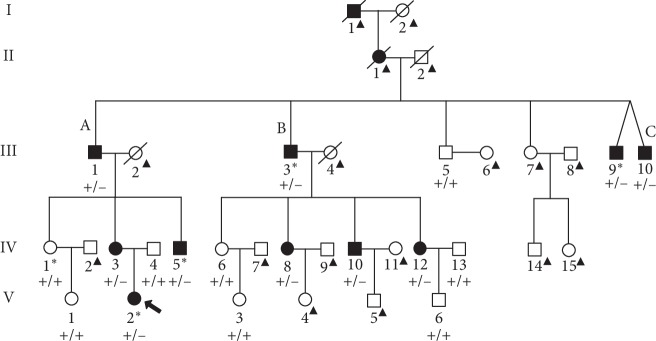
The pedigree structure of the DOA family. Slashes denote deceased family members. The arrow indicates the proband. Squares denote the male family members, and circles denote the female family members. Solid black symbols denote vision loss patients, and open white symbols denote unaffected members. III-9 and III-10 are twins. The number below each member denotes the tag of each patient. The letters A, B, and C above three subfamilies denote their subfamily tags. The individuals marked with an asterisk (*∗*) were analyzed by whole exome sequencing. For individuals marked with a triangle (^▲^), no blood sample was available. Genotypes of the *OPA1* mutation (c.1313A>G, p.D438G) are indicated below each pedigree symbol (+, wildtype allele; −, mutant allele).

The pedigree consisted of 35 family members from 5 generations. There was no consanguineous marriage in this family. The male-to-female ratio in patients with DOA was 7 : 5. These pedigree features together with male-to-male transmissions suggested an autosomal dominant inheritance in this family (Figure 1). Twelve individuals in this family had vision loss (I-1, II-1, III-1, III-3, III-9, III-10, IV-3, IV-5, IV-8, IV-10, IV-12, and V-2), and the others had normal vision. The proband (V-2) and her mother (IV-3) had vision loss; however, the vision of her father (IV-4) was normal. V-2 was born at full term without systemic diseases and bad habits. She was diagnosed as optic atrophy because of poor vision at 3 years old. The patient III-1 had DOA accompanied with left ear hearing loss, and he had drinking history for 60 years. The patient III-3 was diagnosed with rheumatic osteoarthrosis at 30 years old and left femoral head necrosis at 58 years old, and he had smoking and drinking history for more than 35 years. Patients III-9 and III-10 are twin brothers. Patient III-9 had smoking and drinking history for more than 20 years. Patient III-10 was diagnosed with blue and yellow color anomalopia at 20 years old, and he had smoking and drinking history for more than 15 years. Patient IV-5 was treated using intravenous mouse nerve growth factor in 2012, but it was not effective, and he had drinking history for 8 years. Patient IV-10 had smoking and drinking history for more than 15 years. All patients had clear or mild dense lens and normal macula lutea and IOP (less than 21 mmHg). Except that I-1 and II-1 were deceased, clinical characteristics of the other 10 patients with DOA are shown in Table 1. Analysis of the clinical characteristics of the 10 patients showed remarkable variable expressivity, and phenotypic heterogeneity were observed among patients.

### 3.2. Eye Manifestations

**Figure 2:**
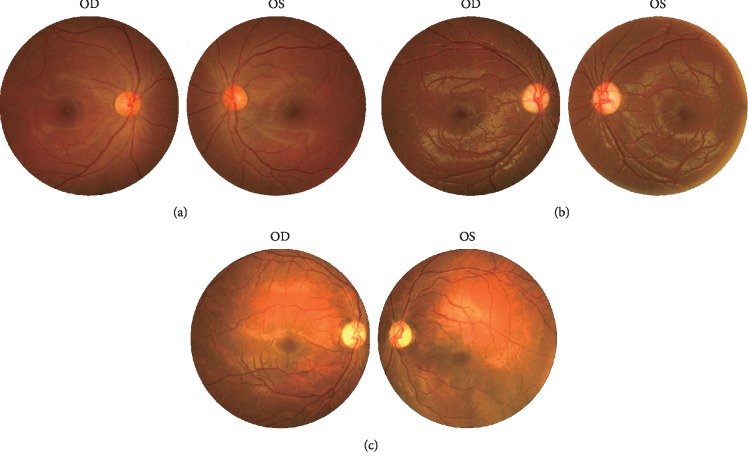
Fundus photography of three members in the DOA family. The fundus photography of an unaffected family member IV-1 (a) and two DOA patients, V-2 (b) and IV-10 (c). The optic discs of (a) are orange, and their borders are clear. The optic discs of (b) are pale in the temporal area. The optic discs of (c) are pale.

**Figure 3:**
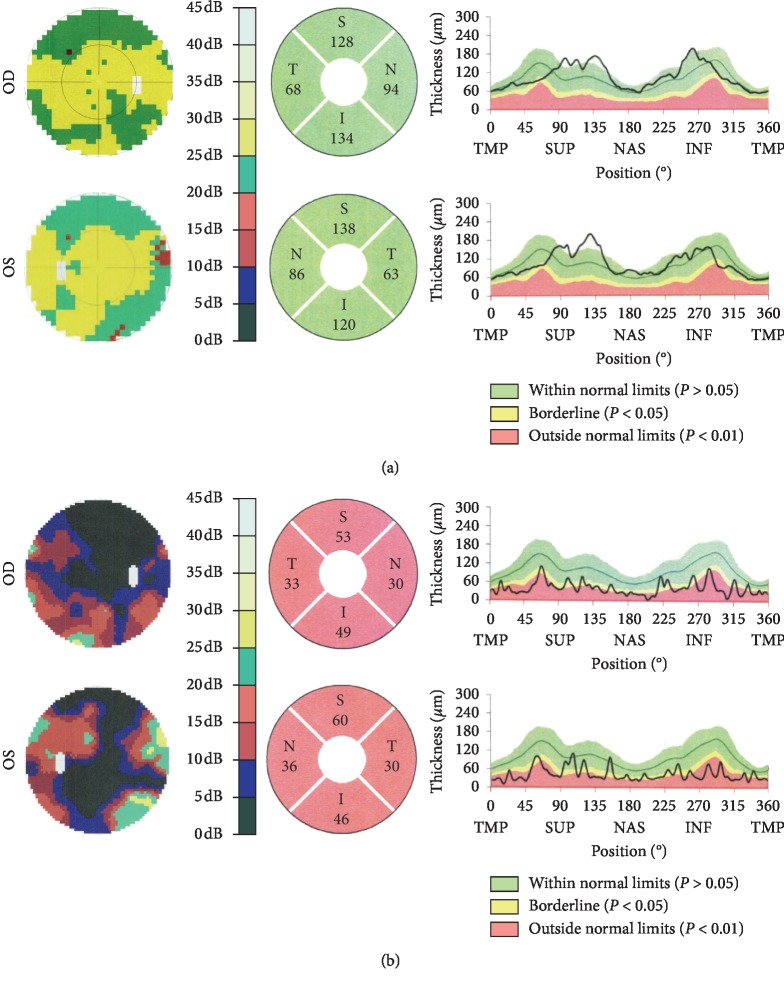
Visual field and OCT exam results of two members in the DOA family. The bilateral visual field (left panel) and OCT exam (middle and right panels) results of an unaffected family member IV-1 (a) and a DOA patient III-9 (b). (a) had normal visual field (dynamic balance test strategy) and normal OCT-RNFL thickness. The range of RNFL thickness for four quadrants was showed in the right panel. (b) had bilateral extensive VFD (central low vision test strategy) and bilateral extensive thinned OCT-RNFL thickness.

**Figure 4:**
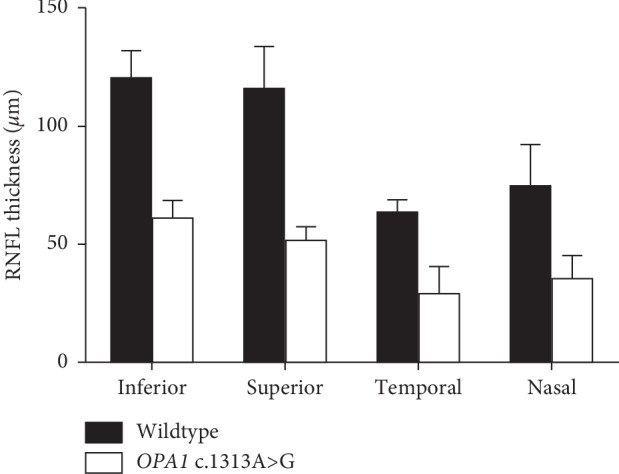
The comparison of RNFL thickness between patients and controls in four quadrants in the DOA family. *X* axis denotes the inferior, superior, temporal, and nasal quadrants. *Y* axis denotes the RNFL thickness (*μ*m). The RNFL thickness of patients was significantly thinner than that of controls, respectively, in inferior, superior, temporal, and nasal quadrants; all *P* < 0.05 (*n* = 16).

**Figure 5:**
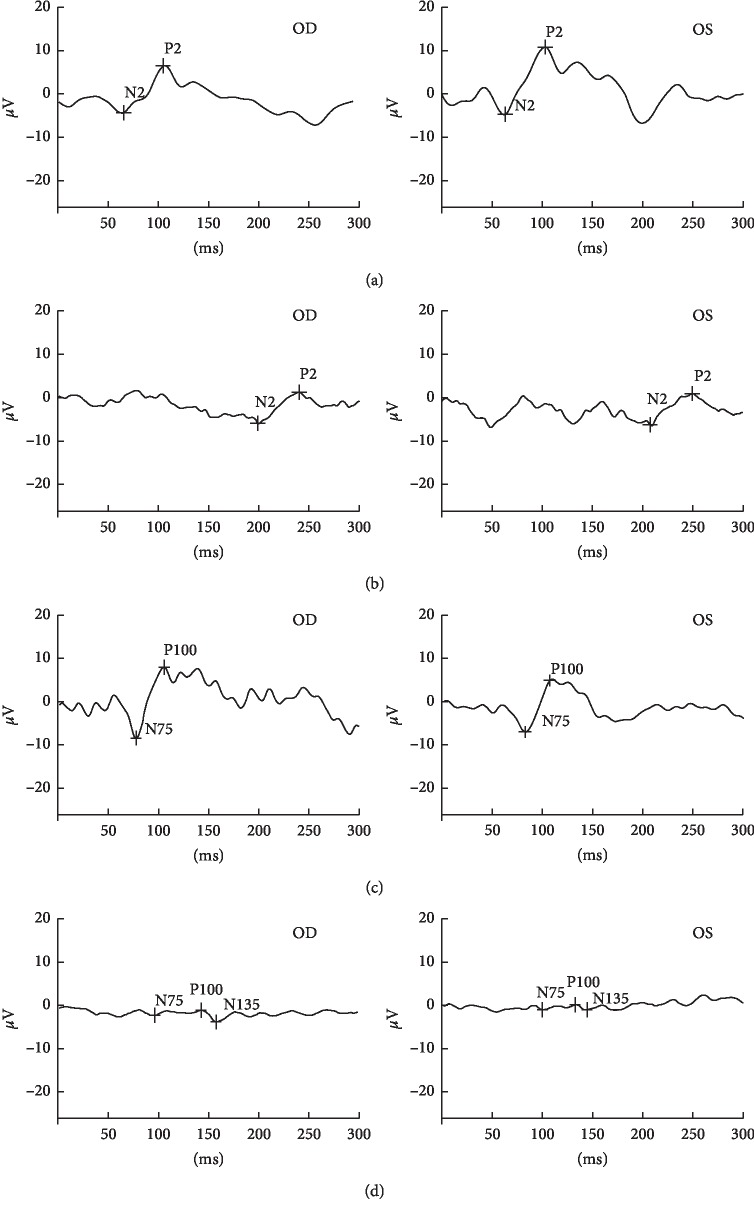
VEP test results of four members in the DOA family, and F-VEP tests were presented. (a) An unaffected family member III-5 showing a normal bilateral F-VEP pattern. (b) A DOA patient IV-5 showing prolonged P-wave latencies and severe lower P-wave amplitudes in both eyes. (c) P-VEP tests were presented in an unaffected family member IV-1, showing a general normal bilateral P-VEP pattern. (d). A DOA patient IV-3 showing a severe P100 amplitude reduction in both eyes.

The severity of vision loss in this family varied tremendously. BCVA ranged from hand movement to 20/28 (Table 1). The BCVA in smokers was significantly lower than in nonsmokers among patients of this family (*P* < 0.05; Table 2). The BCVA in alcohol drinkers was significantly lower than in nondrinkers among patients of this family (*P* < 0.001; Table 2). Fundoscopy suggested full pallor or temporal pallor of optic disc on both eyes (Figure 2). The RNFL thickness in four quadrants of the DOA group was significantly thinner than in unaffected controls by OCT (*Z* = 3.256, *P* < 0.001; *Z* = 3.271, *P* < 0.001; *Z* = 3.259, *P* < 0.001; *t* = 4.863, *P* < 0.05 in superior, inferior, temporal, and nasal quadrants, respectively) (Figures 3 and 4). F-VEP suggested mild-to-severe prolonged P-wave latencies and lower P wave; P-VEP presented mild-to-severe P100 amplitude reduction (Figure 5).

### 3.3. Identification of the *OPA1* Mutation

The WES data of 5 family members (4 patients: V-2, III-3, III-9, and IV-5 and one healthy member: IV-1) captured 99.8%-99.9% of all exons in the genome. The average read depth was over 114×. 99.6% targeted regions were covered for 10×, and 98.4% targeted regions were covered for 20× times. A total of 392,379 single nucleotide polymorphisms (SNPs) and 58,595 indels were called from the WES data (Table 3). After variant filtering, 1,900 SNPs and 884 indels were obtained for further analysis. After checking the segregation of variants with the disease status in the family, a missense mutation (c.1313A>G, p.D438G) in the *OPA1* gene located at 3q29 screened by WES was the best candidate mutation or likely responsible for the disease in this family. The mutation was predicted to be pathogenic (SIFT = 0.005, possibly damaging; Polyphen-2 = 1.0, possibly damaging; MutationTaster = 1.0, disease causing; and CADD = 28.9) and was extremely conservative (GERP score: 5.8).

### 3.4. Verification of the *OPA1* Mutation

**Figure 6:**
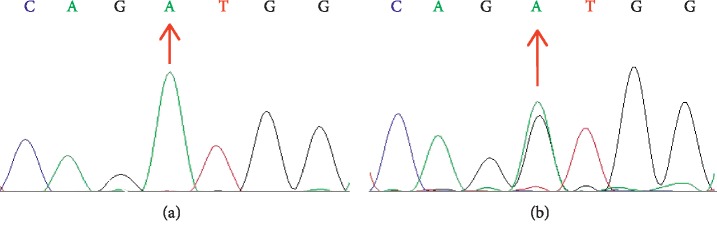
Sanger sequencing chromatogram showing a portion of the sequenced region of *OPA1*. (a) A wildtype (arrow) of a control. (b) A heterozygous missense mutation (c.1313A>G, arrow) of a patient.

The *OPA1* mutation (c.1313A>G, p.D438G) was verified using PCR and Sanger sequencing in all 4 DOA patients who underwent WES. Further sequencing of the other 12 family members who had DNA samples in this pedigree confirmed segregation of the mutation with the disease status in this family (Figure 6). No mtDNA mutation in *LHON* was found in all family members who had DNA samples (data not shown).

## 4. Discussion

In this study, we identified an *OPA1* missense mutation (c.1313A>G, p.D438G) responsible for the disease in a Chinese family affected with DOA (Figure 1). The *OPA1* gene is located at 3q28-29 and has 31 exons and 8 transcript variants. *OPA1* encodes a mitochondrial dynamin-related GTPase, which is localized to the inner mitochondrial membrane and helps regulate mitochondrial stability and energy output. This protein is also required for maintaining the integrity of cristae junctions and preventing the leakage of cytochrome C [20–22]. The OPA1 protein shares several structural features with dynamins, including a GTPase domain containing three consensus: GTP-binding sequences and a dynamin segment, a middle domain, and a GTPase effector domain containing a coiled-coil region. Among these, the GTPase domain and the C-terminal coiled-coil domain are most common damaged areas of DOA [23].

The c.1313A>G (p.D438G) mutation of *OPA1* identified in this study is for the first time found in the Chinese population, although it was initially reported in an Iranian DOA family [24]. It is hypothesized that the p.D438G mutation leads to misalignment of GTP in the GTP-binding pocket of the GTPase domain of OPA1. As a consequence, GTP hydrolysis will be impaired, resulting in reduced mitochondrial function. Initially, carriers cope well with the ensuing energy deficiency [24]. However, the highly energy-demanding and delicate retinal ganglion neurons eventually suffer gradual damage due to the continuous lack of sufficient energy, leading to the insidious onset of visual loss, a characteristic for optic atrophy [24]. We concur with their protein structural modeling that this mutation leads to the mitochondrial fusion failure, resulting in the RGC death. Intriguingly, Pesch et al. found a different missense mutation at the same site (c.1313A>T, D438V) in two German DOA families [25]. Both mutations significantly impair the GTPase domain and the dynamin-related core region. Understanding how these mutations cause the RGC death may provide valuable information for developing a therapeutic treatment for DOA.

The clinical characterization of the DOA family in this study revealed a remarkable phenotypic heterogeneity. First, the age of onset varied among patients in this family. For example, patients of subfamily A developed DOA in childhood, whereas the disease was diagnosed in adolescence in patients of subfamily B (Figure 1; Table 1). In particular, patient III-10 in subfamily C was diagnosed at the age of 5 years, while his twin brother III-9 was diagnosed at the age of 13 years (Figure 1; Table 1), suggesting that the age of onset varied by 8 years between the twins. Second, some patients had severely impaired vision of finger counting or hand movement (e.g., III-1, III-3, III-9, III-10, and IV-3), while some other patients had relatively good vision of 20/28 (e.g., IV-12). Third, most of the patients in this family had smoking and/or drinking habits. The smokers/alcoholics had poorer vision, severe optic atrophy, and VEP abnormality (Table 1). Patients who were smokers had significantly lower BCVA than nonsmokers. Similarly, the BCVA of alcohol drinkers was significantly lower than that of nondrinkers in patients (Table 2). Since no matching in gender and age between the groups (smokers *vs.* nonsmokers and alcohol consumers *vs.* nonalcohol consumers) was possible in this family, these factors might have contributed to the differences in BCVA of the DOA patients. Tobacco and alcohol were well documented as risk factors for toxic and nutritional optic neuropathy, and recent studies had shown that tobacco consumption triggered disease manifestation in LHON [26]. We found tobacco and alcohol consumption to the detriment of DOA, which was in agreement with previous reports that tobacco smoking plays an important role in the pathogenesis of many posterior segment eye diseases [27, 28]. Since tobacco contains free radical-generating oxidants, the oxidant can cause damage or even death of RGC and loss of retinal nerve fibers through ischemia or high oxidative stress mechanism. Tobacco smoking or chronic nicotine poisoning may cause neurotoxicity of RNFL, resulting in reduced thickness of the retina [28]. Not only smoking can deteriorate optic neuropathy, drinking is also one of the risk factors of optic nerve. Tufan et al. found that chick embryos exposed to different concentrations of ethanol resulted in both retinal degeneration and optic nerve hypoplasia in a dose-dependent manner [29]. Previous studies have also suggested that the combination of two risk factors, smoking and drinking, could contribute to the development of optic neuropathy [30]. Due to the significant heterogeneity in clinical manifestations among patients in the family in this study, we proposed that environmental factors and living habits such as smoking and drinking could lead to the phenotype heterogeneity of DOA.

In clinics, the diagnosis of DOA needs to be differentiated from LHON, which is also a hereditary optic neuropathy. LHON is more common in young males and may have unilateral progression of vision loss. The fundus exam shows congested or hydropic optic disc borders and dilated peripheral capillaries in the early stage. Pale temporal optic disc and bilateral central or paracentral scotoma may be found in the later stage [31]. No gender difference was indicated in the family in this study (male-to-female ratio of 7 : 5), and the symptoms were observed in both eyes. The fundus exam of proband V-2 showed temporal pale optic disc without any congestion and no dilated peripheral capillaries. These clinical features support the initial diagnosis of DOA. Genetic tests can assist in differential diagnosis of DOA from LHON. LHON is a maternally inherited disease due to mitochondrial DNA mutation. The most common mutations are found in the *NADH dehydrogenase subunit* (*ND*) *4*, *ND1*, and *ND6* genes. Mutations in *ND4 m.11778G>A*, *ND1 m.3460G>A*, and *ND6 m.14484T>C* account for 50%–70%, 6%–25%, and 10%–15% of LHON patients, respectively [32–34]. In this study, we did not find any mtDNA mutations in *LHON* patients. In contrast, DOA is an autosomal inherited optic neuropathy, mainly caused by mutations of the *OPA1* gene. To date, five genes (*OPA1*, *OPA3* [35], *AFG3L2* [36], *DNM1L* [37], and *WFS1* [38]) have been identified as disease-causing genes for nonsyndromic DOA. In addition, six genetic loci have been reported to be responsible for DOA, including *OPA2* (Xp11.4-p11.21), *OPA4* (18q12.2-q12.3), *OPA5* (22q12.1-q13.1), *OPA6* (8q21-q22), *OPA7* (11q14.1), and *OPA8* (16q21-q22) [2, 6]. Among them, *OPA1* is the leading gene of DOA [39]. More than 280 *OPA1* mutations have been reported to responsible for DOA (http://mitodyn.org; updated on December 30, 2018).

The drug treatment with idebenone and gene therapy such as rAAV2/2-ND4 has been shown to be effective for LHON in clinical trials [40, 41]. Unfortunately, no clinically proven treatment option for DOA is available yet. Avoiding harmful environmental factors such as tobacco and alcohol exposure is highly recommended to ameliorate the condition. Translational research for DOA is entering an accelerated phase with the availability of animal models, and a variety of pharmacological and genetic therapies are being developed [42, 43]. Further functional studies are needed to provide further insights into this inherited eye disease.

## 5. Conclusions

In summary, we have identified a missense mutation in *OPA1* responsible for the disease in a Chinese family affected with DOA. Clinical manifestations of the patients were heterogeneous in this family. This is the first report on the c.1313A>G (p.D438 G) mutation of *OPA1* identified in a Chinese DOA family.

## Figures and Tables

**Table 1:** Clinical characteristics of patients with DOA in the Chinese family.

Subfamily	A				B				C	
Patient	III-1	IV-3	IV-5	V-2	III-3	IV-8	IV-10	IV-12	III-9	III-10
Gender	M	F	M	F	M	F	M	F	M	M
Age at enrollment/age at diagnosis (years)	77/10	28/3	23/9	8/3	63/14	35/15	33/18	29/16	52/13	52/5
Smoking/drinking	−/+	−/−	−/+	−/−	+/+	−/−	+/+	−/−	+/+	+/+
BCVA(OD,OS)	HM,HM	FC,FC	20/500,	20/125,20/125	HM,HM	20/100,	20/333,	20/30,20/28	FC,FC	HM,HM
			20/500			20/100	20/667			
Pupil (mm)	5 *∗* 5	4.5 *∗* 4.5,	4.5 *∗* 4.5,	Normal	5.5 *∗* 5.5,	Normal	3.5 *∗* 3.5,	Normal	4.5 *∗* 4.5,	6 *∗* 6,
		RAPD(+)	RAPD(+)		RAPD(+)		RAPD(+)		RAPD(+)	RAPD(+)
Optic disc	Pale	Pale	Pale	Temporal pale	Pale	Temporal pale	Pale	Temporal pale	Pale	Pale
Lab tests	Family member IV-3: mildly abnormal F-VEP (OU); severely abnormal P-VEP (OU). Family member IV-5: moderately abnormal F-VEP (OU); extensively thinned RNFL thickness of OCT (OU); extensive VFD (OU). Family member V-2: mildly to moderately abnormal F-VEP (OS); moderately to severely abnormal P-VEP (OU); extensively thinned RNFL thickness of OCT (OU).				Family member III-3: moderately to severely abnormal F-VEP (OS); extensively thinned RNFL thickness of OCT (OU). Family member IV-10: temporal thinned RNFL thickness of OCT (OU); extensive VFD (OU).				Family member III-9: moderately to severely abnormal F-VEP (OU); extensively thinned RNFL thickness of OCT (OU); extensive VFD (OU); yellow color anomalopia. Family member III-10: moderately to severely abnormal F-VEP (OS); blue and yellow color anomalopia (OU).	

M: male; F: female; BCVA: best corrected visual acuity; OU: eye, both; OD: eye, right; OS: eye, left; HM: hand movement; FC: finger counting; RAPD: relative afferent pupillary defect; F-VEP: flash visual evoked potential; P-VEP: pattern visual evoked potential; OCT: optical coherence tomography; RNFL: retinal nerve fiber layer; VFD: visual field defect.

**Table 2:** The comparison of BCVA (logMAR) between smokers/alcohol drinkers and nonsmokers/nondrinkers in patients of the DOA family.

Patients	Eyes (*n*)	BCVA (mean ± SD)	*t*	*P*
Smokers	8	1.950 ± 0.427	2.521	0.021
Nonsmokers	12	1.200 ± 0.761		

Alcohol drinkers	12	1.917 ± 0.438	4.270	<0.001
Nonalcoholdrinkers	8	0.875 ± 0.658		

BCVA: best corrected visual acuity; SD: standard deviation.

**Table 3:** Total number of variants called from the whole exome sequencing data.

Individual	V-2	III-3	III-9	IV-5	IV-1	Total
SNPs	187,398	195,216	192,720	174,243	185,388	392,379
Indels	23,794	25,222	25,231	22,832	23,801	58,595

SNP: single nucleotide polymorphism; indel: insertion/deletion.

## Data Availability

The data used to support the findings of this study are included within the article.
